# Mobile app validation: a digital health scorecard approach

**DOI:** 10.1038/s41746-021-00476-7

**Published:** 2021-07-15

**Authors:** Ramy Sedhom, Michael J. McShea, Adam B. Cohen, Jonathan A. Webster, Simon C. Mathews

**Affiliations:** 1 Memorial Sloan Kettering Cancer Center, Department of Medicine, New York, NY USA; 2 Next Generation Care Delivery, National Health Mission Area, The Johns Hopkins University Applied Physics Lab (APL), Laurel, MD USA; 3 Health Technologies, National Health Mission Area, The Johns Hopkins University Applied Physics Lab (APL), Laurel, MD USA; 4 Johns Hopkins Medicine, Department of Neurology, Baltimore, MD USA; 5 Johns Hopkins Medicine, Department of Oncology, Division of Hematology, Baltimore, MD USA; 6 Johns Hopkins Medicine, Department of Internal Medicine, Division of Gastroenterology, Baltimore, MD USA

**Keywords:** Health policy, Patient education

## Abstract

While digital health solutions continue to grow in number and in complexity, the ability for stakeholders in healthcare to easily discern quality lags far behind. This challenge is in part due to the lack of a transparent and standardized approach to validation. Evaluation of mobile health applications (apps) is further burdened by low barriers to development and direct-to-user marketing, leading to a crowded and confusing landscape. In this context, we investigated the pragmatic application of a previously described framework for digital health validation, the Digital Health Scorecard, in a cohort of 22 popular mobile health oncology apps. The apps evaluated using this framework performed poorly, scoring 49.4% across all evaluation criteria as a group. Performance across component domains varied considerably with cost scoring highest at 100%, usability at 56.7%, technical at 37.3%, and clinical at 15.9%. satisfaction of prospectively determined end-user requirements derived from patient, family, and clinician consensus scored 37.2%. While cost outperformed consistently and usability was adequate, the results also suggested that apps suffered from significant technical limitations, were of limited clinical value, and generally did not do what end users wanted. These large gaps further support the need for transparent and standardized evaluation to help all stakeholders in healthcare improve the quality of mobile health.

## Introduction

Digital health technologies, including mobile health applications (apps), hold great potential to improve American health and healthcare^1^—there is nearly ubiquitous use of smartphones by Americans and an ever-growing and increasingly sophisticated suite of health apps. These apps are providing a wide range of medical functions that span the care continuum from prevention to diagnosis to care management^2,3^. They are also increasingly demonstrating an impact on clinical outcomes with recent successes in cancer^4^, cardiac health^5^, and mental health^6^. The adoption of these digital solutions is further amplified by their accessibility, low cost, and personalized features. In addition, their ability to provide practical functions such as health education, tracking of symptoms and side effects, appointment management, and social support make them compelling healthcare tools.

While the range and number of healthcare apps directly accessible to patients continue to grow, it remains difficult to reliably discern quality, which is hindered by the lack of standardized evaluation and validation processes^7,8^. Historically, only a fraction of health apps are appropriate for use^9^, which is concerning since patients are influenced by the health information they discover on their own^8^. In addition, common online metrics (e.g., star rating or the number of downloads) do not correlate with clinical utility or validity^10^. While identifying quality in mobile apps is an issue for all stakeholders in medicine, it is more acutely experienced by patients and physicians who directly seek these solutions. As the technology capabilities underpinning these apps increase, so do the potential risks. This trend makes the need for nimble evaluation even greater. Several recent studies have provided a broad overview of mobile apps in oncology^11,12^ and others have focused on provider^13,14^ and patient perspectives^15^ in general; however, very few have focused on the pragmatic implementation of app evaluation. Moreover, widely cited generalized mobile app evaluation frameworks, like the Mobile App Rating Scale (MARS)^16^, are underdeveloped in the area of clinical appropriateness. The App Quality Assessment Tool or Health-Related Apps (AQUA)^17^ combines MARS with Enlight^18^, an assessment tool that does incorporate therapeutic concepts. However, this tool, as with its component frameworks, does not include key technical elements beyond privacy and security of data. The THESIS approach^19^ uses the domains of transparency, health content, technical content, security/privacy, usability, and subjective ratings. However, it is does not incorporate clinical evidence. While these frameworks have advanced quality assessment, a pragmatic quality assessment tool that incorporates end-user requirements as well as technical, usability, and clinical dimensions is still needed.

This study’s primary objective was to test the practical application of a previously reported validation framework^7^ for mobile apps with results presented within a digital health scorecard. Oncology apps were selected as the primary focus area since cancer patients represent a diverse population and are likely to seek out health information^19^. In addition, offerings in oncology are diverse and vast, with over one thousand mobile apps available^20,21^.

## Results

### Overall summary

**Fig. 1:**
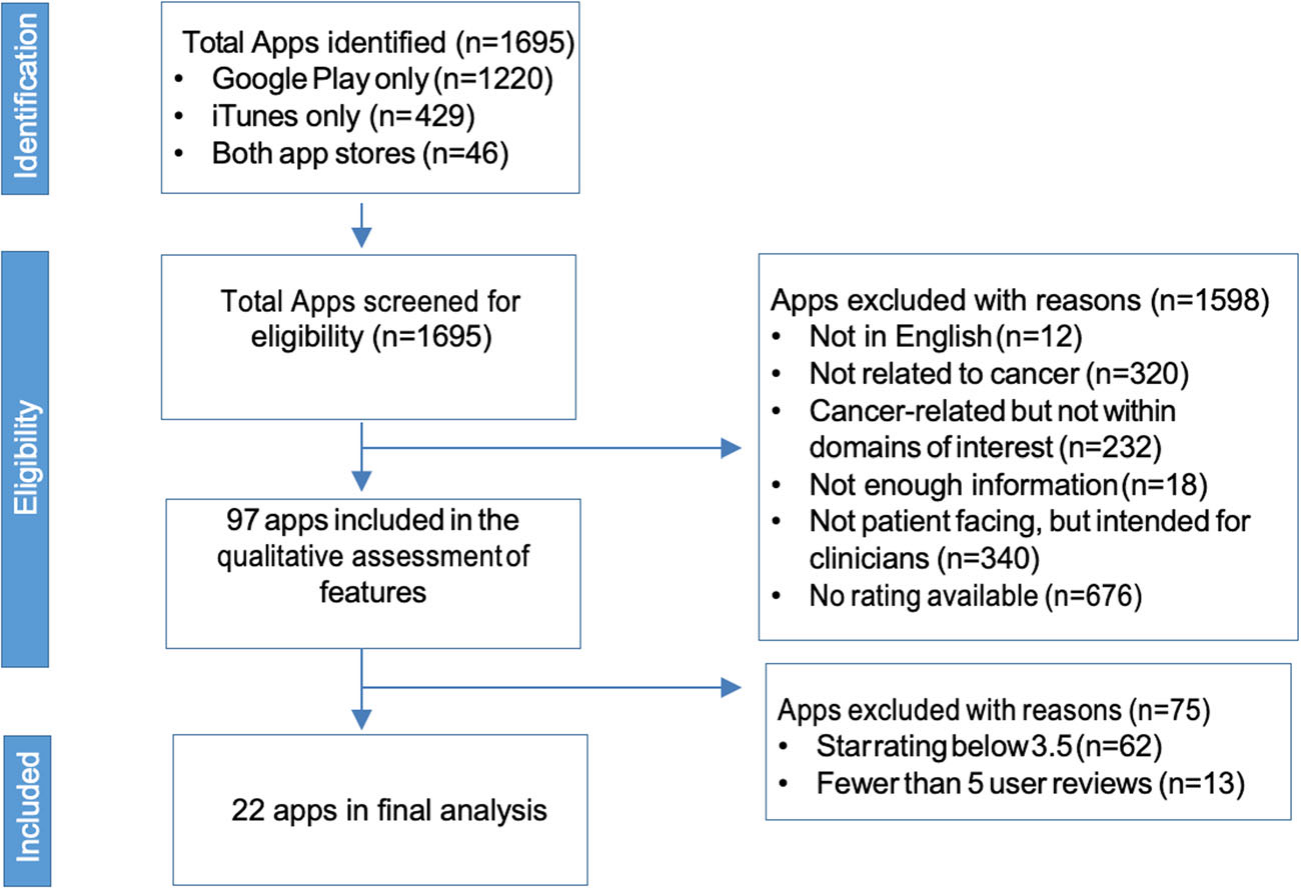
Mobile app selection process. Out of 1695 apps identified, 22 apps were eligible based on exclusion criteria outlined in figure.

The process of identification and inclusion of apps is outlined in Fig. 1. We examined a total of 18 Apple and 4 Android apps. Six were focused on education, 5 on prevention, 6 on social support, and 5 in our sample (*n* = 20, 91%) made no clinical claim, and 2 (9%) had misleading/erroneous information. The mean total score for all apps evaluated across all domains and end-user requirements was 49.4%. Individual domain scores varied with a mean score of 37.3% for technical, 15.9% clinical; 56.7% for usability, 100% for cost, and 37.2% for end-user requirements.

**Fig. 2:**
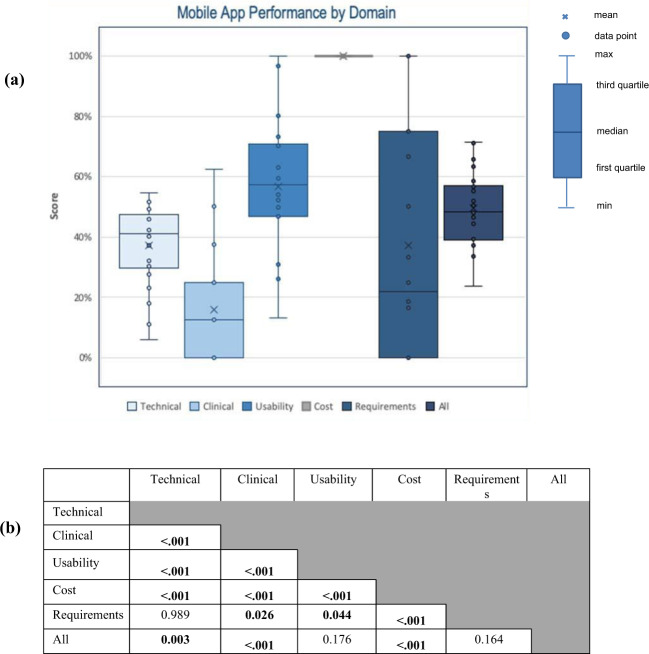
Mobile app performance by domain. **a** Mobile app performance across domains of technical, clinical, usability, cost, and end-user requirements. **b** Statistical comparison of performance among domain areas and composite score with *p*-values shown.

There was considerable inter-app variation across domain and end-user performance (Fig. 2a). The majority of inter-category score differences were statistically significant (Fig. 2b). When excluding the cost domain, the mean total score across all apps was 36.8%.

### Focus areas

**Fig. 3:**
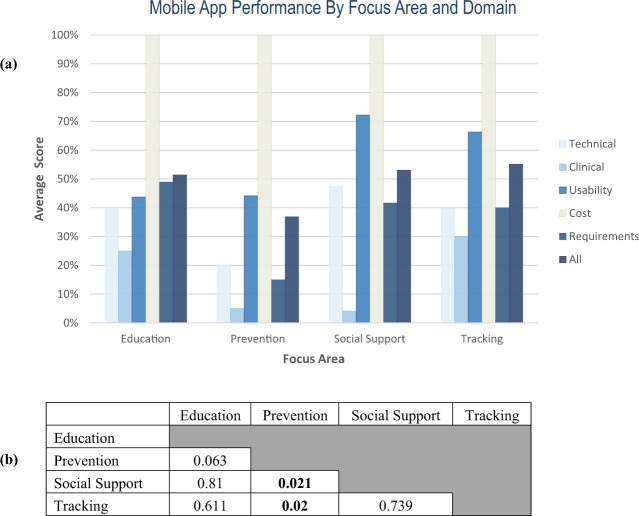
Mobile app performance by focus area and domain. **a** Mobile app performance by focus area and domain areas. Mean scores across technical, clinical, usability, cost, and end-user requirement domains are represented in bar graphs. **b** Statistical comparison among focus areas.

**Fig. 4:**
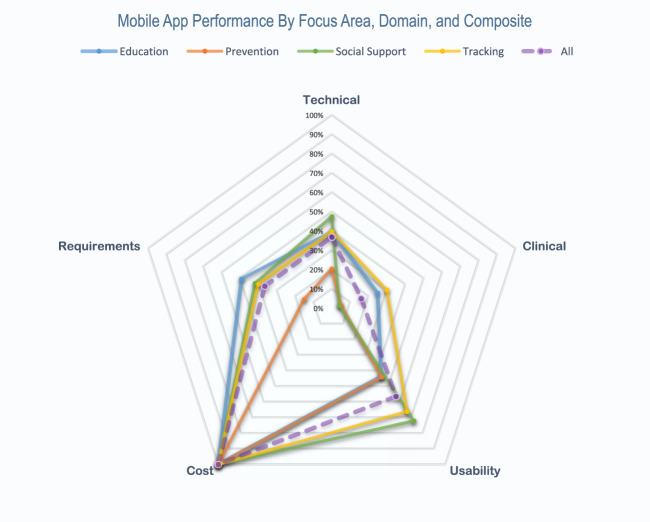
Mobile app performance by focus area, domain, and composite. Distance from center to each point of the pentagon indicates performance in each respective domain domain. Colors represent app focus areas and dashed purple line represents composite performance of all apps.

Performance by focus area also varied with mean scores of 51.4% for education, 36.9% for prevention, 53.1% for social support, and 55.2% for tracking with significant heterogeneity across domains and end-user requirement scores as well (Fig. 3a) with most statistical differences appreciated when comparing against the prevention focus area (Fig. 3b). Compared to the composite average domain scores for all apps, the prevention category underperformed in all domains except cost, which was equal across all focus areas (Fig. 4). Tracking apps outperformed the all-app composite in all domains and end-user requirements, while education apps outperformed the average in all domains and end-user requirements, except usability. Social support apps underperformed in the clinical domain but were otherwise above average.

### Domain areas

Apps performed best in the domain of cost where all scored a maximum value of 100%. All apps tested were free. Usability was the second highest performing domain, with high subdomain scores in visual design and notifications, alerts, and alarms. The mean scores in these two categories were 68 and 73%, respectively. The technical domain’s highest scoring subdomain was privacy with an average score of 48% across all apps. The remaining three subdomains scored in the low 30% range. The clinical domain had the lowest overall performance—its highest subdomain score was credibility which averaged 31%. The two apps that were scored “X” for misleading/false information were assigned a numeric value of 0 so as not to artificially inflate the total cohort domain score by excluding them. Education apps scored the highest with respect to performance on end-user requirements with an average score of 58%, followed by social support at 42%, tracking at 40%, and prevention at 15%.

### Figure descriptions

**Fig. 5:**
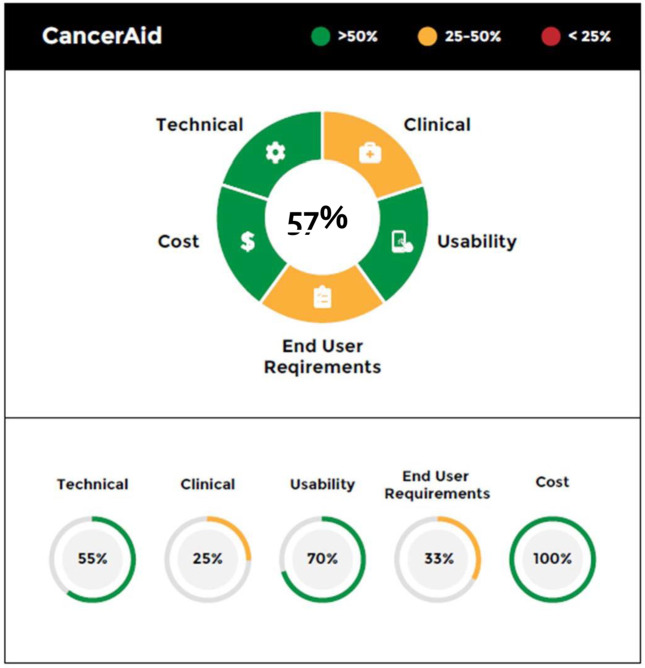
Digital health scorecard, individual summary. Individual domains are depicted in a segmented wheel and color coded based on performance scores. The overall score for the app is depicted in the center of the segmented wheel.

**Fig. 6:**
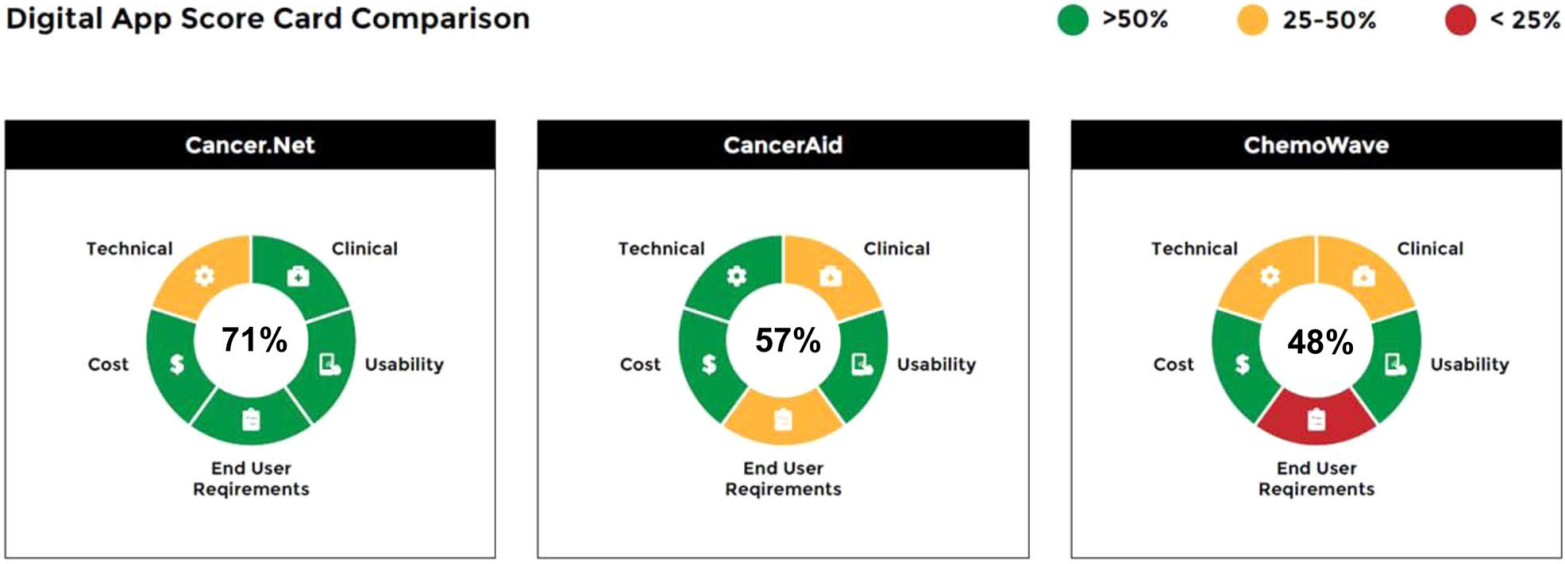
Digital health scorecard, comparison view. A digital health scorecard comparison for three apps is depicted, with individual domains represented in segmented wheels, and color coded based on performance scores.

**Fig. 7:**
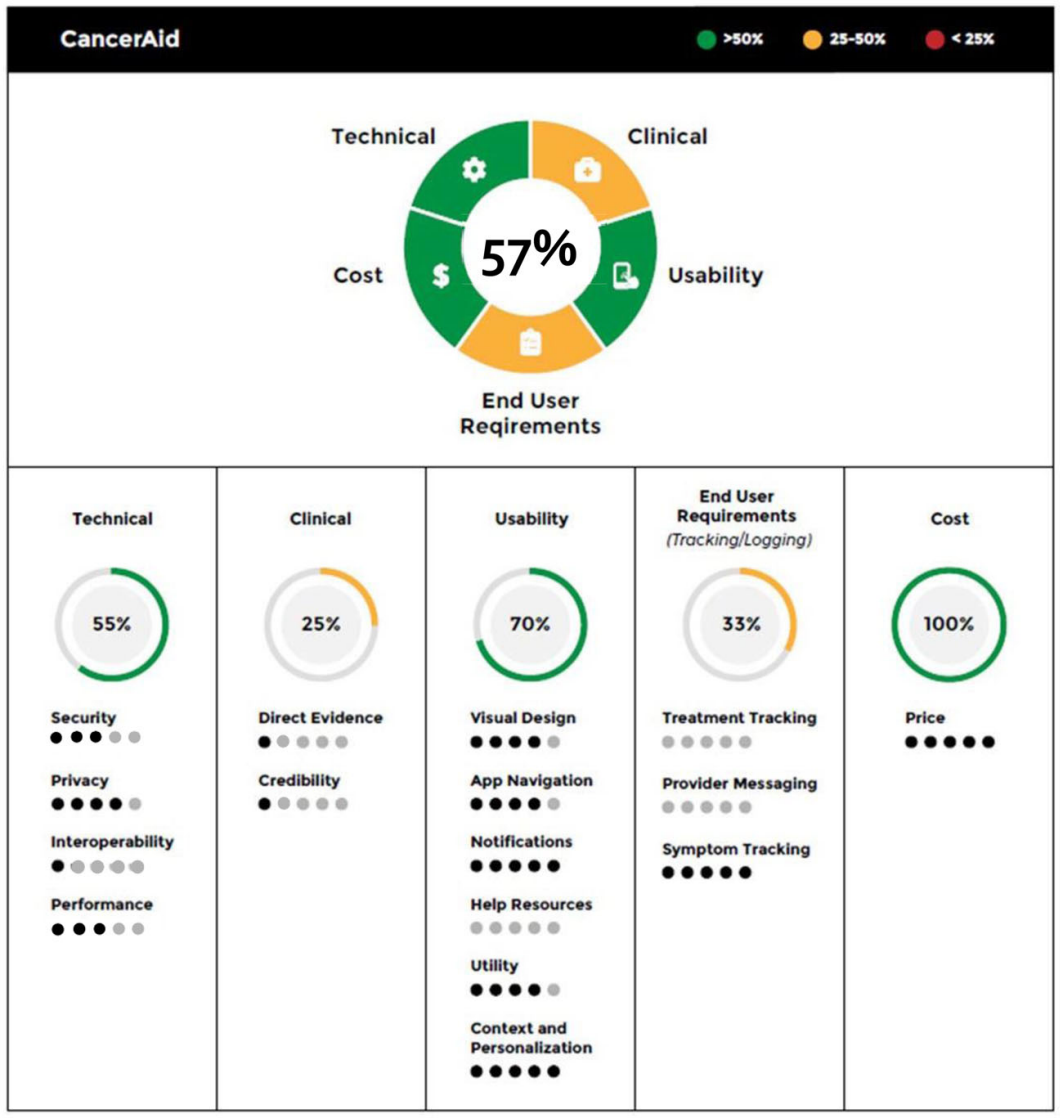
Digital health scorecard, granular view. Number in wheel at top indicates overall performance with individual domain performance summarized in segmented portions of wheel. Performance by domain is detailed below the wheel with overall domain score indicated with subdomain performance included below.

Figures 5–7 provide different visual representations of the digital health scorecard which represent composite assessments based on the scoring above. Figure 5 is a visual representation for the individual app, CancerAid. The 4 domains and end-user requirement assessment are depicted in a segmented wheel, and color coded based on performance scores. In this example, CancerAid had the following performance scores: technical 55%, clinical 25%, usability 70%, end user requirements 33%, and cost 100%. The overall score for the app was 57%, and depicted in the center of the segmented wheel. A digital health scorecard comparison for three apps is illustrated in Fig. 6.

Figure 7 offers a more granular description of performance across each individual domain.

Subdomain scores were normalized and rounded to the nearest whole number and assigned zero to five bullets depending on performance in that subdomain. As a result of rounding, the fraction of completed bullets per domain does not always equal the exact percentage score for that domain. However, normalization was performed to facilitate easier comparisons among domains and subdomains. In the example provided, CancerAid (a tracking app) allows users to track symptoms, but does not meet the end-user requirements necessary for tracking treatment or messaging providers. Similarly, in the technical domain, CancerAid had strong security, privacy, and performance features, but interoperability was limited.

## Discussion

The quality of mobile health apps that physicians and patients encounter varies markedly. Our findings demonstrated that popular oncology apps generally did not meet high quality standards across multiple domains and end-user requirements. These broad deficiencies call into question whether widespread use of these solutions is appropriate or beneficial. There was significant variability in performance across individual domains and focus areas. That the apps evaluated were popular suggests that individual domain attributes may have attracted users instead of complete solutions or that users may not be aware of app quality metrics.

Within the cohort evaluated in this study, mobile apps scored poorly in the technical and clinical domains and in end-user requirements—that is, they had major technical deficiencies, showed little clinical benefit, and did not satisfy what the users wanted them to do. With all apps being free, the cost domain did not serve as a useful discriminator of value or quality. However, the lack of cost may in part explain the popularity of these solutions. Usability scored the second highest, which may reflect the broader maturity of app design relative to attributes in other domains and may explain app utilization. For example, basic aspects of functionality, including navigation and notifications, have been rigorously iterated upon in other industries and have reached a competitive standard that is not seen in other domains. This may be a function of these attributes being directly consumer facing, whereas elements such as privacy and security, which are also broadly applicable to other industries but less visible to users, were low for most oncology apps. Apps also generally scored poorly with interoperability and performance measures. This highlights the ongoing need to more broadly implement minimum technical standards in these areas.

Clinical performance was low and driven by the low number of apps making any clinical claim. This finding likely reflects the present state of mobile apps in oncology and highlights a significant gap in the current solution landscape. In addition to most apps not reaching a high clinical bar, some made misleading or false claims. This latter scenario presents a potentially dangerous situation particularly as apps become more sophisticated through advancements in technology such as image and movement sensors and machine learning based artificial intelligence. This presents an opportunity for medical societies, academic centers, and other trusted entities to partner with developers to produce trustworthy, high-quality, and evidence-based solutions.

The vast majority of apps did not adequately satisfy end-user requirements. The cohort average was 37.2% which highlights the significant discrepancy between what is being built for patients and physicians and what these stakeholders actually want apps to do. Incorporating these stakeholder perspectives in the product development lifecycle may help improve the overall functionality and effectiveness of the product. These results and the findings more generally demonstrated the wide disparity between what is popular and what is high quality. An initial step in bridging this gap is making the gap transparent to all stakeholders. Without systematic and transparent evaluation and a clear determination of end-user needs, patients, physicians, and other stakeholders have no reliable way to assess app quality. Our results also demonstrate that users cannot discriminate quality by using assessments available through the traditional digital gatekeepers—app stores.

There are key limitations of the framework and results described. The oncology setting and the resulting focus areas do not reflect the entire range of mobile health apps available. Thus, this study does not represent a comprehensive assessment of all oncologic use cases, settings, and needs. In addition, the end-user requirements reflected the priorities from a sample of oncology conditions and were collected from a small group of patients and providers—other stakeholders and larger, more diverse groups were not represented. In the setting where the end-user perspective from the appropriate clinical and solution specific context is characterized by real-world data, this information could serve as a more representative substitute for the design groups. Thus, assessing both more conditions (and apps) and a larger, more diverse patient population would allow more generalizable conclusions. However, the narrower approach in this study allowed an assessment of the unique needs and use cases for the apps available for evaluation. Another limitation was the inability to discriminate quality using the cost domain. This challenge may continue to persist in the general evaluation of mobile apps where price, set up, and maintenance costs are generally low or non-existent. However, other areas of health technology are unlikely to have this same degree of homogeneity, and direct integration of mobile apps into broader care delivery solutions may introduce more discriminatory capabilities in the cost domain.

There is considerable variability in how the health technology evaluation approach herein could be executed and adapted to other technologies and applications. For example, we chose criteria based on standard, best clinical practice and made subjective decisions regarding the thoroughness of evaluation (i.e., number and type of evaluation criteria). The approach we chose represented a balance between efficiency and comprehensiveness. Financial, human, and time resources will also impact the criteria used. As a result, in different stakeholder contexts, the range and depth of evaluation could vary. We recommend establishing consensus-based tiers of evaluation for each domain to maintain consistency and transparency. A minimum set of criteria may also be useful to ensure the validity of the assessment.

Regardless, making these criteria transparent and widely available will be key to achieving consensus and driving higher standards for mobile health app development.

The digital health scorecard and its underlying evaluation framework offer a pragmatic, requirements-driven, and impact-focused approach to evaluating the digital health landscape. Our study provides a practical demonstration for implementing standards-based assessment that produces accessible and meaningful results for clinicians and patients. While future research is still needed to validate the digital health scorecard approach, this study is a formative step in advancing a theoretical concept to a real-world application. The road to validating digital health will take resources, collaboration, and time. We believe an important next step is to establish an evaluation process that reflects the real-world needs of stakeholders in healthcare. The digital health scorecard represents one possible path.

## Methods app selection process

**Fig. 8:**
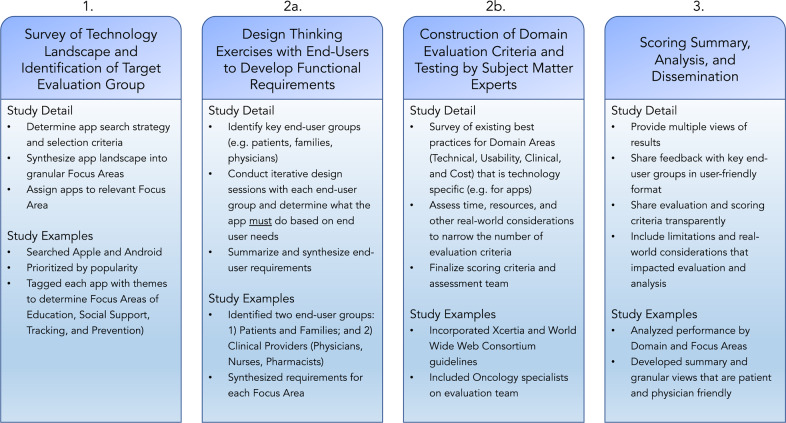
Summary of digital health scorecard approach. Phases of evaluation are depicted, beginning with technology target assessment, followed by determination of requirements, development of assessment criteria, and finally, scoring and analysis.

A summary of the digital health scorecard approach below is outlined in Fig. 8. We first used a structured search query using 6 search terms to identify cancer related apps on US Android Google Play and Apple iTunes store. The search terms used were “cancer”, “cancer support”, “cancer care”, “breast cancer”, “prostate cancer”, and “best cancer apps”. Apps were eligible to be included in this review if they met all of the following criteria: (1) apps that aimed to support patients, (2) apps related to cancer, (3) apps with a star rating, (4) apps that were in English, and (5) apps that were targeted into one of our four domains of interest. These domains of interest were identified as most important through stakeholder engagement with patients and clinicians in an iterative process, and include prevention, management, social support, and education. This resulted in a preliminary review of 97 apps by a multidisciplinary team. We aimed to include apps that were used by a large number of patients or individuals, and further excluded apps that had a star rating below 3.5 and/or fewer than 5 user reviews. We then identified 22 oncology apps for substantive evaluation based on the composite ranking of the highest ratings and number of reviews as of July 15, 2019. Information about this apps retrieved from the search was entered into an electronic spreadsheet. The information entered included name of the app, name of the developer, cost, app store or stores in which the app was available, and the search term or terms that retrieved the app. *App Evaluation Process* App validation was then performed by applying a previously described framework^7^, which encompassed evaluation across the following domains: technical, clinical, usability, and cost. In addition, per the framework, end-user requirements were identified and assessed. The app focus areas and end-user requirements were determined and validated through design thinking sessions with key stakeholders detailed below. Each app was downloaded and tested by two independent app developers who completed the technical, usability, and end-user requirement evaluations for each app. A group of three physicians performed the clinical domain evaluation—including two hematology–oncology specialists. Purchase price was recorded at the time of download for all apps. The domains of evaluation are summarized briefly below. A detailed summary of the individual elements of evaluation are provided in Supplement 1.

Technical validation encompassed evaluation of security, privacy, interoperability, and performance^7^. The scoring for the technical domain included 25 individual criterion, which were scored 0 (none) to 2 (full) for a maximum domain score of 50.

Clinical validation referred to an assessment of evidence supporting any clinical claims using an approach based on the GRADE system^22^. Scoring for the clinical domain included the two subdomains of direct evidence and credibility. For the former, if the technology did not make a clinical claim to impact a health outcome, a score of 0 was given. If the technology provided any false or misleading clinical information, a score of “X” was given. If a clinical claim was made, a maximum of 4 points were given if the technology was supported by a randomized clinical trial or meta-analysis, and a minimum score of 1 if the clinical claim was made based on expert opinion alone. Credibility was approximated by appraising any recognition by known affiliations, associations, or endorsements. Credibility scores range from a maximum of 4 (produced by recognized medical society or organization) to a minimum of 0 (not produced or recognized by a medical society, organization, institution, team, or individual). A maximum score for this domain was 8.

Usability assessment focused on whether the mobile technology was patient-oriented and the degree to which the app design followed good principles for positive user experience. The app was evaluated to determine ease of use for its intended purpose, the effort required to complete tasks, data entry burden, and whether users were allowed to control preferences such as notifications when appropriate. This perspective aligned with the World Wide Web Consortium (W3C) guidelines, which summarized a user-centered design process (UCD)^23^. The key objectives state apps should be useful in helping users achieve their goals, effective (i.e., producing results with minimal user error), learnable (i.e., easy and intuitive to use), and likeable (i.e., enjoyable to use). Usability frameworks have been in place since the early days of software applications, most notably the System Usability Scale (SUS)^24^, which has been used for 35 years. However, usability assessed with SUS does not include consideration of mobile app design specific concepts, or address user likeability. For our usability assessment, two digital health-specific evaluation frameworks were analyzed against the overarching objectives W3C, the Xcertia Guidelines^25^, and the Node.Health User Experience (UX) Framework^26^, both published in 2019. A pragmatic approach was taken based on incorporating the strongest elements of each framework, augmented with essential elements of consumer-based frameworks^27^. The resulting scoring for the usability domain was divided into six subdomains (visual design and readability; app navigation; notifications, alerts, alarms; help resources; utility; and context and personalization). A total of 22 individual items were scored, with a range from 0 to 5 (maximum score = 110).

Cost in the validation framework incorporated a number of variables including face-value price, time for set up and use, additional training, and maintenance expenses. However, this domain was simplified to price alone since the remaining variables did not apply or did not add any additional discriminatory value.

End-user requirements were determined through design thinking exercises conducted with end users, including patients with cancer, their caregivers, physicians, nurses, and pharmacists^28^. These sessions determined the attributes most important to the end user for each focus area (education, social support, tracking, prevention). Education apps were scored on whether content was personalized and whether it was presented in easy to understand terms. Social support apps were scored on their ability to connect patients with community resources and an online community of similar patients. Tracking apps were scored on their ability to track individual treatment plans, their ability to connect patients with their healthcare team, and their ability to track symptoms over time. Prevention apps were expected to provide personalized risk assessments and risk mitigation techniques. Only the requirements in the focus area applicable to each app was scored.

### Scoring and statistical approach

A full schematic of the scoring sheet is provided in Supplement 1. The total score for each app, which is shown as a percentage of total possible points, provides equal weight to each of the evaluation domains (technical, clinical, usability, cost, and end-user requirements). Likewise, subdomains within each of the evaluation domain were normalized and treated with equal weights. This allowed scoring to be independent of how many evaluation criteria were used for each subcategory. For example, the composite technical score was based on the weighted sum of normalized underlying subdomain scores: security (20), privacy (14), interoperability (10), and performance (6). Each subdomain score was calculated and weighted equally when determining the total average for the technical domain. Statistical comparisons of mean scores between domains categories and between focus areas were conducted using the two-tailed, unpaired *t*-test.

### Reporting summary

Further information on research design is available in the Nature Research Reporting Summary linked to this article.

## Supplementary information

Supplementary Data 1

Supplementary Information

Reporting Summary

## Data Availability

Upon reasonable request, our team will provide aggregated data used in the findings of this study.
