# Supplementary material for: Mobile app validation: a digital health scorecard approach
Source: NPJ Digit Med. 2021 Jul 15;4:111. doi: 10.1038/s41746-021-00476-7 (PMC8282811; doi:10.1038/s41746-021-00476-7)
Supplement: Supplementary file 2 — Supplementary Information [file 41746_2021_476_MOESM2_ESM.pdf]

Guideline	Domain	Title	Assessment Criteria	Points (0 = none; 1 = partial 2 = full)	Section Total
JHU1-01	Technical	Security	Is all data encrypted (both in flight and at rest)?	0 to 2	Add up individual scores
JHU1-02	Technical	Security	Does the application require a login?	0 to 2	
JHU1-03	Technical	Security	Does the application support 3rd party identity providers (e.g. Google, Facebook, etc.)	0 to 2	
JHU1-04	Technical	Security	Does the app provide a logout mechanism?	0 to 2	
JHU1-05	Technical	Security	Is sensitive data stored on the cloud?	0 to 2	
JHU1-06	Technical	Security	Has the application been certified by any 3rd party security vendors?	0 to 2	
JHU1-07	Technical	Security	Does the application send sensitive data via e-mail?	0 to 2	
JHU1-08	Technical	Security	Does the application prevent brute-force login attempts by having lockout mechanism?	0 to 2	
JHU1-9	Technical	Security	Does the application include features of strong authentication such as password strength, security questions, and tokens?	0 to 2	
JHU1-10	Technical	Security	Does the application have a password recovery function?	0 to 2	
JHU2-01	Technical	Privacy	Does the application require necessary access to phone components (e.g. Camera, Microphone, etc.)?	0 to 2	Add up individual scores
JHU2-02	Technical	Privacy	Is the application provided by an independent developer or business entity?	0 to 2	
JHU2-03	Technical	Privacy	Does the application have a data use agreement / terms of service?	0 to 2	
JHU2-04	Technical	Privacy	Does the application request consent for use of data?	0 to 2	
JHU2-05	Technical	Privacy	Does the application give the user the ability to control what data is shared?	0 to 2	
JHU2-06	Technical	Privacy	Does the application have a privacy policy?	0 to 2	
JHU2-07	Technical	Privacy	Does the application state compliance with HIPAA (1) and/or GDPR (2)	0 to 2	
JHU3-01	Technical	Interoperability	Does the application provide a means to export user's data (e.g. PDF (1), CSV (2), JSON (2)?	0 to 2	Add up individual scores
JHU3-02	Technical	Interoperability	Does the application support FHIR APIs for patient data?	0 to 2	
JHU3-03	Technical	Interoperability	Does the application have multiple form factors (e.g. phone, laptop, tablet)?	0 to 2	
JHU3-04	Technical	Interoperability	Is the app available in iOS and Android formats?	0 to 2	
JHU3-05	Technical	Interoperability	Does the app have connectivity support (e.g. Bluetooth)	0 to 2	
JHU4-01	Technical	Performance	Does the application crash close regularly?	0 to 2	Add up individual scores
JHU4-02	Technical	Performance	Does the application have no perceptible delays in screen loading or log-in?	0 to 2	
JHU4-03	Technical	Performance	Is there documentation the application content been updated within the past 3 months (2) or 6 months (1)?	0 to 2	

Guideline	Domain	Title	Assessment Criteria	Points	Section Total
JHU1-01	Clinical	Direct Evidence	Does the technology make a clinical claim to impact a health outcome? If yes, see below	0 = no and proceed to Credibility	Add up individual scores
JHU1-02	Clinical	Direct Evidence	Is the clinical claim made by the technology supported by randomized control trial or meta-analysis?	0 = no; 4 = yes	
JHU1-03	Clinical	Direct Evidence	Is the clinical claim made by the technology supported by non-randomized control trial or cohort study?	0 = no; 3 = yes	
JHU1-04	Clinical	Direct Evidence	Is the clinical claim made by the technology supported by case-control studies; cross-sectional surveys; case reports?	0 = no; 2 = yes	
JHU1-05	Clinical	Direct Evidence	Is the clinical claim made by the technology supported by expert opinon alone?	0 = no; 1 = yes	
JHU1-06	Clinical	Direct Evidence	Is there any evidence to suggest clinical claim made by the technology is false or misleading?	0 = no; X = yes	
JHU2-01	Clinical	Credibility	Produced by Recognized medical institution or organization	0 = no; 4 = yes	Add up individual scores
JHU2-02	Clinical	Credibility	Produced by Recognized medical individual or team	0 = no; 3 = yes	
JHU2-03	Clinical	Credibility	Endorsed by recognized medical institution/organization	0 = no; 2 = yes	
JHU2-04	Clinical	Credibility	Endorsed by recognized medical individual/team	0 = no; 1 = yes	
JHU2-05	Clinical	Credibility	None of the above	0 = yes	

Guideline	Title	Performance Requirement	Points	1 to 5 Scale
JHU1-01	Visual Design & Readability	Interfaces should be flexible to operate in both orientations for different screen form factors	0 or 1	Add up individual scores
JHU1-02	Visual Design & Readability	App should minimize need to scroll, and when scrolling is necessary users should be able to clearly identify when screens extend beyond the scroll line.	0 or 1	
JHU1-03	Visual Design & Readability	When possible, reduce the probability of data entry error by providing users with selectable options rather than requiring text entry.	0 or 1	
JHU1-04	Visual Design & Readability	Apps should should present information in easy to read format, e.g. chunked text vs. large paragraphs and appropriate text size	0 or 1	
JHU1-05	Visual Design & Readability	Text should avoid use of jargon or acronyms that may not be familiar to users, particularly for lay users without clinical knowledge.	0 or 1	
JHU2-01	App Navigation	Users should be able to easily identify where they are in the app and how to navigate to different destinations, including reversing actions. The navigational path should be logical, predictable, and easy to follow.	0 to 2	Add up individual scores
JHU2-02	App Navigation	Navigating to primary tasks (from one area of the app to another) should require a minimal number of taps, swipes, or screens.	0 or 1	
JHU2-03	App Navigation	The app's main menu should be easily locatable and identifiable and labeled intuitively.	0 or 1	
JHU2-04	App Navigation	On-boarding is completed in a timely manner and the user clearly understands the capabilities of the application after on-boarding	0 or 1	
JHU3-01	Notifications, Alerts, Alarms	Users should be given the choice to opt out of automatic non-critical notifications and alerts.	0 to 2	Add up individual scores
JHU3-02	Notifications, Alerts, Alarms	User's should be able to set reminders where app content is prescriptive for actions	0 = no; 1 = N/A; 2 = yes	
JHU4-01	Help Resources & Support	Apps should have an easy-to-locate help section that consolidates all information intended to assist the user.	0 to 2	Add up individual scores
JHU4-02	Help Resources & Support	Help features and informational links should be imbedded in the app when users may be likely to need them (?), with appropriate use of pop-ups or links.	0 to 2	
JHU4-03	Help Resources & Support	Human support is provided in addition to digital support	0 or 1	
JHU5-02	Utility	Single tasks should not require more than 1-2 screens and should minimize scrolling	0 to 2	Add up individual scores
JHU5-03	Utility	Information and functions needed for a particular task or decision making (JTD) is grouped together in a single location	0 or 3	
JHU6-01	Context & Personalization	Data appropriate for historical reference and trending should be stored and available to the user	0 or 1	Add up individual scores
JHU6-02	Context & Personalization	Content and screens are personalized based on individual user situation and needs	0 to 2	
JHU6-03	Context & Personalization	Must leverage data to add value to the user. 0 - Presentation only. 1 - Incorporates context to provide value from data. 2 - Predictive & AI usage of data	0 or 2	

Guideline	Domain	Title	Assessment Metric	Value	Section Total
JHU1-01	Cost	Price	What is the initial purchase price?	Dollar amount	Total Cost
JHU1-02	Cost	Price	Are there recurring fees or additional purchases required?	NA	
JHU1-03	Cost	Price	Are there recurring fees or additional purchases optional?	NA	
JHU1-04	Cost	Price	What is the estimated cost to maintain technology over 1 year?	NA	
JHU2-01	Cost	Time	How much time does it take to set up?	NA	Total Time
JHU2-02	Cost	Time	How much time does it take to use?	NA	
JHU2-03	Cost	Time	Is there additional training required?	NA	

Guideline	Category	Focus Area	End User Type	Assessment Metric	Points (0 = none; 1 = partial 2 = full)	Section Total
JHU1-01	User Requirement	Education	Provider; Patient/Family	The app must have educational content that is personalized for me	0 to 2	Total
JHU1-02	User Requirement	Education	Patient/Family	The app content should be presented in layman's terms/ easy to understand	0 to 2	
JHU2-01	User Requirement	Social Support	Provider; Patient/Family	The app must connect to community resources that can aid me	0 to 2	Total
JHU2-02	User Requirement	Social Support	Provider; Patient/Family	The app must connect w/ an online community of similar patients - forums , social media , direct chat	0 to 2	
JHU3-01	User Requirement	Tracking/Logging	Provider; Patient/Family	The app must track my treatment plan including ; meds, appointments, other interventions	0 to 2	Total
JHU3-02	User Requirement	Tracking/Logging	Patient/Family	The app must have ability to connect/ message my healthcare team/ provider	0 to 2	
JHU3-03	User Requirement	Tracking/Logging	Provider; Patient/Family	The app must log my symptoms and view them historically	0 to 2	
JHU4-01	User Requirement	Prevention	Provider; Patient/Family	The app must provide personalized risk assessments	0 to 2	Total
JHU4-02	User Requirement	Prevention	Provider; Patient/Family	The app must provide risk mitigation techniques	0 to 2	
